# Transcription factor retention through multiple polyploidization steps in wheat

**DOI:** 10.1093/g3journal/jkac147

**Published:** 2022-06-24

**Authors:** Catherine E B Evans, Ramesh Arunkumar, Philippa Borrill

**Affiliations:** Department of Crop Genetics, John Innes Centre, Norwich Research Park NR4 7UH, UK; School of Biosciences, University of Birmingham, Birmingham B15 2TT, UK; Department of Crop Genetics, John Innes Centre, Norwich Research Park NR4 7UH, UK; Department of Crop Genetics, John Innes Centre, Norwich Research Park NR4 7UH, UK

**Keywords:** transcription factors, evolution, polyploidy, *Triticum aestivum* L. (wheat), gene balance hypothesis, Shared Data Resource

## Abstract

Whole-genome duplication is widespread in plant evolutionary history and is followed by nonrandom gene loss to return to a diploid state. Across multiple angiosperm species, the retained genes tend to be dosage-sensitive regulatory genes such as transcription factors, yet data for younger polyploid species is sparse. Here, we analyzed the retention, expression, and genetic variation in transcription factors in the recent allohexaploid bread wheat (*Triticum aestivum* L.). By comparing diploid, tetraploid, and hexaploid wheat, we found that, following each of two hybridization and whole-genome duplication events, the proportion of transcription factors in the genome increased. Transcription factors were preferentially retained over other genes as homoeologous groups in tetraploid and hexaploid wheat. Across cultivars, transcription factor homoeologs contained fewer deleterious missense mutations than nontranscription factors, suggesting that transcription factors are maintained as three functional homoeologs in hexaploid wheat populations. Transcription factor homoeologs were more strongly coexpressed than nontranscription factors, indicating conservation of function between homoeologs. We found that the B3, MADS-M-type, and NAC transcription factor families were less likely to have three homoeologs present than other families, which was associated with low expression levels and high levels of tandem duplication. Together, our results show that transcription factors are preferentially retained in polyploid wheat genomes although there is variation between families. Knocking out one transcription factor homoeolog to alter gene dosage, using TILLING or CRISPR, could generate new phenotypes for wheat breeding.

## Introduction

Gene duplication plays a major role in the evolution of genetic and phenotypic diversity and in speciation events in eukaryotes (Lynch and Conery 2000; Van de Peer, Maere, *et al.* 2009). Ancient whole-genome duplications (WGD) are observed throughout the angiosperm plant phylogeny and occurred at the base of major clades such as the seed plants, core eudicots, and monocots (Van de Peer, Fawcett, *et al.* 2009; Tasdighian *et al.* 2017). Following WGD, most gene duplicates are eventually lost from the genome by fractionation over the course of millions of years (Freeling 2009). Nevertheless, a significant portion of duplicates are retained in multiple plant lineages (Lloyd *et al.* 2014; Li *et al.* 2016). Duplicated genes may maintain their original function, for example if increased dosage (number of gene copies) is advantageous (e.g. Dhar *et al.* 2014; Tumen-Velasquez *et al.* 2018) or if selection favors genetic buffering (Nowak *et al.* 1997). Alternatively, gene copies may diverge in function through subfunctionalization, for example mediated by complementary degenerative mutations in each copy (Force *et al.* 1999), or evolve new and distinct functions through neofunctionalization (Ohno 1970). However, these mechanistic explanations cannot account for the observation that across a wide range of plant species, the genes retained as duplicates belong to specific functional classes, encoding transcription factors (TFs) and components of protein complexes (Blanc and Wolfe 2004; Seoighe and Gehring, 2004; Maere *et al.* 2005; Freeling 2009).

Addressing this gap, the gene balance hypothesis proposes that dosage-sensitive genes tend to be retained as duplicates (Birchler *et al.* 2005; Birchler and Veitia 2007). This hypothesis explains the observation that the loss of genes after WGD is nonrandom and certain classes of gene are preferentially retained including genes involved in regulatory interactions or in protein complexes that are dosage sensitive (Blanc and Wolfe 2004). Conversely, these dosage-sensitive genes are less frequently found in segmental duplications in which they would upset the dosage balance with interacting partners (Maere *et al.* 2005), in contrast to WGD where their interacting partners would also be duplicated.

Studies across multiple angiosperms have revealed that TFs, a major type of dosage-sensitive regulatory gene, tend to be retained as duplicates after WGD for millions of years, as predicted by the gene balance hypothesis (Lloyd *et al.* 2014; Li *et al.* 2016). In a comparative study of 37 sequenced angiosperm genomes, Li *et al.* (2016) found that duplicate genes that originated at the Cretaceous-Paleogene boundary ∼50–70 million years ago (mya), when a large number of WGD events occurred (Van de Peer, Fawcett, *et al.* 2009), were enriched for TFs. However, these angiosperm-wide studies focused on relatively old WGD events >5 mya, whilst more recent WGD events which have occurred in individual lineages and are found in several major crop species are less well studied, perhaps due to a lack of genome sequences. Preferential retention of dosage-sensitive genes such as TFs has been observed in the young polyploid *Tragopogon miscellus* which underwent WGD only ∼80 years before (Buggs Richard et al. 2012). This study used a limited number of loci; therefore, there remains a need to understand the effects of recent (<5 mya) WGD at a genome-wide scale. Results from Brassica allotetraploids formed 7,500–12,500 years ago indicate that preferential retention of dosage-sensitive genes is also observed in this short time span (Zhang *et al.* 2021) but work in additional species is needed to broaden these conclusions. A further gap in our knowledge is the variability within an individual species for duplicate gene retention, which can be investigated using genetic variation data now available for polyploid crops such as wheat.

Hexaploid bread wheat evolved from two hybridization and WGD events: allotetraploid wild emmer wheat (*Triticum turgidum* ssp. *dicoccoides*) was formed ∼0.4 mya when the A genome progenitor *Triticum urartu* hybridized with the B genome progenitor species (Feldman and Levy 2012). The allotetraploid emmer was domesticated and hybridized with the D genome progenitor *Aegilops tauschii* ∼10,000 years ago to form hexaploid bread wheat (*T.* *aestivum* L.; Dubcovsky and Dvorak 2007). This two-step recent history of WGD events has resulted in >50% of genes being present with three homoeologous copies in bread wheat [International Wheat Genome Sequencing Consortium (IWGSC) 2018]. Previous studies in wheat have shown that 58% of NAC TFs and 63% of MIKC-type MADS-box TFs have three homoeologs (Borrill *et al.* 2017; Schilling *et al.* 2020), but a systematic study has not been carried out to establish whether the preferential retention of TFs is observed across all TF families in this recent polyploid.

In this study, we investigated whether these two recent WGD events resulted in the preferential retention of TFs in hexaploid wheat, as would be predicted by the gene balance hypothesis. Using the curated expression data available for wheat, we explored alternative hypotheses about TF retention, for example whether divergent gene expression was associated with homoeolog retention. Moreover, since genetic variation in several TFs has been instrumental in wheat adaptation during domestication including the free-threshing gene *Q* (Simons *et al.* 2006) and the vernalization gene *VRN1* (Yan *et al.* 2003), we examined the natural variation in TF homoeologs observed in wheat. Specifically, we examined the propensity of wheat TFs to be retained as functional copies without deleterious mutations at a population level. Hence our study addresses not only an evolutionary question about the retention of TFs in young polyploids but also provides insight into TF expression diversity and genetic variation which lays a foundation for future research and breeding.

## Materials and methods

### Annotation of TFs in wheat and progenitor species

Peptide sequences for all transcript isoforms in the RefSeqv1.1 gene annotation of *T.* *aestivum* cv. Chinese Spring [International Wheat Genome Sequencing Consortium (IWGSC) 2018] were downloaded from EnsemblPlants (Howe *et al.* 2020). The file was divided into three parts to contain <50,000 sequences per file and TFs were annotated in each file using iTAK online v1.6 (Zheng *et al.* 2016). In cases where different transcript isoforms were assigned to different TF families (23 out of 6,128 genes), the family assigned to the longer transcript isoform was retained (Supplementary Table 1). Peptide sequences for genes in the *A.* *tauschii* assembly (Luo *et al.* 2017) were downloaded from EnsemblPlants, divided into six smaller files, and annotated using iTAK online v1.6. Again, when different transcript isoforms were assigned to different TF families (186 out of 2,120 genes), the family assigned to the longer transcript isoform was retained (Supplementary Table 2). In general, discrepancies between TF families were due to one isoform being truncated, with the truncated isoform lacking a protein domain that allowed a more specific TF family to be assigned to the longer isoform. Coding sequences for the longest isoforms of genes in the *T.* *urartu* genome (Ling *et al.* 2018) were downloaded from http://www.mbkbase.org/Tu/ (access date 2nd June 2020) and annotated using iTAK online v1.6 (Supplementary Table 3). TF annotations for *T.* *turgidum* ssp. *dicoccoides* cv. Zavitan (Avni *et al.* 2017) were downloaded from the iTAK database (update 18.12; Zheng *et al.* 2016; Supplementary Table 4).

### Identification of diads in tetraploid wheat and triads in hexaploid

Diads in tetraploid wheat are a set of homoeologous genes that have a 1:1 correspondence across the A and B genomes. Similarly, triads in hexaploid wheat are homoeologous genes that have 1:1:1 correspondence across the A, B, and D genomes. Homoeologs in *T. turgidum* ssp. *dicoccoides* were obtained from Avni *et al.* (2017) and filtered to only retain 1:1 homoeologs by removing “singleton” and “hit2homolog” (i.e. paralog) groups (Supplementary Table 4). Homoeologs in *T. aestivum* were downloaded from EnsemblPlants Biomart for the RefSeqv1.1 gene annotation using only high confidence gene models. Only one2one homoeologs (assigned by EnsemblPlants) were retained. There were 20,393 triads corresponding to 61,179 genes (56.7% of genes) (Supplementary Table 1). Only high confidence genes from the RefSeqv1.1 annotation were used in all subsequent hexaploid wheat analyses.

### Adjusting for the effect of gene loss in tetraploid wheat on hexaploid wheat triad numbers per TF family

In order to adjust for the differences in triad proportions between TF families observed in hexaploid due to the varying proportions in diads in tetraploid wheat, we calculated the normalized percentage of genes in triads:

$$\mathrm{Normalized}\;\mathrm{percentage}\;\mathrm{of}\;\mathrm{genes}\;\mathrm{in}\;\mathrm{triads}=\frac{\%\;\mathrm{of}\;\mathrm{genes}\;\mathrm{in}\;\mathrm{triads}\;\mathrm{in}\;\mathrm{hexaploid}\;\mathrm{wheat}}{\%\;\mathrm{of}\;\mathrm{genes}\;\mathrm{in}\;\mathrm{diads}\;\mathrm{in}\;\mathrm{that}\;\mathrm{TF}\;\mathrm{family}}.$$

For example if 60% of genes were in triads in hexaploid, but only 80% of genes were in diads in tetraploid, the normalized value will be 75%—i.e. 75% of the potential triads were formed because we have accounted for the 20% which were already missing in tetraploid.

### Correlation of expression levels per family to homoeolog retention in triads

To measure the gene expression level of each TF family, we used RNA-seq data from 15 different tissues and developmental stages from Chinese Spring (Choulet *et al.* 2014). These included tissues from seedling roots and shoots through to grain 30 days after anthesis. We downloaded gene expression data in transcripts per million (tpm) for this dataset from expVIP (www.wheat-expression.com (access date 2nd June 2020); Borrill *et al.* 2016; Ramírez-González *et al.* 2018). We calculated the mean expression level for each gene across the 15 tissues and then calculated the median expression level for each TF family. We fitted a linear regression model between log(median expression level per TF family) and the percentage of TFs in triads in the family.

### Correlation of tandem duplication per family to homoeolog retention in triads

For all TF genes, we defined tandem duplicates as genes which were adjacent in the genome assembly according to their gene IDs ± 3 genes in either direction (gene IDs increase by 100 for adjacent genes in this genome assembly). We allowed one or two genes between tandem duplicates because a tandem duplication event may have occurred capturing a TF and non-TF in the same duplication event. Each nearby duplicate was counted as one tandem duplication event (i.e. a cluster of three TF genes would be counted as two tandem duplication events), and the total number of tandem duplication events was divided by the total number of genes in each TF family to calculate the percentage of tandem duplicated genes per TF family. We fitted a linear regression model between the percentage of genes which are tandem duplicates per TF family and the percentage of TFs in triads in the family. We repeated our analysis only considering ±2 genes (with one gene between them) or ±1 gene (with no gene between them) as tandem duplicates.

### Calculation of homoeolog similarity of expression per family

Using the same data from 15 different tissues and developmental stages from Chinese Spring, we filtered to only keep triads where at least one homoeolog was expressed >0.5 tpm in one tissue (calculated as the mean value of two biological replicates), consistent with previous studies [International Wheat Genome Sequencing Consortium (IWGSC) 2018; Ramírez-González *et al.* 2018]. To account for differences in expression level between TFs and non-TFs, we normalized the expression level of each triad per tissue to sum to 1 as in Ramírez-González *et al.* (2018) before calculating the standard deviation of expression level between homoeologs. For 58 out of 19,391 triads (0.3%), the TF family was inconsistent between homoeologs (e.g. MYB and MYB-related) so the family assigned to two of the three homoeologs was retained. A Mann–Whitney test was used to determine whether the standard deviation within TF triads was different from non-TF triads for each tissue.

### Calculation of homoeolog coexpression per family

To calculate the Pearson’s correlation between the three homoeologs, we used the same data from 15 different tissues and developmental stages from Chinese Spring. We filtered to only keep triads where at least one homoeolog was expressed >0.5 tpm in one tissue (calculated as the mean value of two biological replicates), and triads where all three homoeologs were expressed (tpm > 0 in at least one tissue). The Pearson’s correlation was calculated between homoeologs within a triad in a pairwise fashion (A vs B, B vs D, A vs D) and the three correlations were plotted for each triad. To calculate the median Pearson’s correlation for TF triads and non-TF triads, the Pearson’s correlation values were Z transformed using DescTools v0.99.44 (Signorell Aema 2021) before obtaining the median, then back-transformed to reduce bias (Corey *et al.* 1998).

As an alternative measure of coexpression, we used information about module assignment from a Weight Gene Co-expression Network Analysis (WGCNA) across 850 wheat RNA samples from a wide range of tissues and developmental stages [Langfelder and Horvath 2008; International Wheat Genome Sequencing Consortium (IWGSC) 2018]. The coexpression network was built using RefSeq v1.0 annotation (downloaded from https://urgi.versailles.inra.fr/download/iwgsc/IWGSC_RefSeq_Annotations/v1.0/). To enable compatibility with our TF annotation which was carried out using RefSeq v1.1 annotation, only genes which were 99% identical with >90% coverage from v1.0 to v1.1 were included in this analysis. To calculate the percentage of triads with homoeologs in the same module only triads in which all three homoeologs had a module assigned, excluding module 0, were considered. Module 0 largely contains genes with invariable expression patterns between samples (Ramírez-González *et al.* 2018).

### Analysis of single nucleotide polymorphism variation data

To investigate the types of single nucleotide polymorphisms (SNPs) in wheat TFs, we used exome capture data of 811 hexaploid wheat landraces and cultivars representing global genetic diversity (He *et al.* 2019). Filtered and imputed SNPs (∼3 million) were downloaded in May 2021 from http://wheatgenomics.plantpath.ksu.edu/1000EC/.

We selected SNPs in genes in triads and used the Ensembl Variant Effect Predictor (VEP v99.2) to predict the effect of SNPs on these genes (McLaren *et al.* 2016). From an input of 529,066 SNPs in triad genes, VEP output 1,146,195 SNP effects. We selected 216,285 SNPs predicted in the coding sequence of the canonical transcript of a triad gene. Using R, we filtered to exclude: SNPs which were also splice region variants; missense variants without Sorting Intolerant from Tolerant (SIFT) scores; and SNPs with >25% missing calls. 210,578 SNPs remained (97% of unfiltered SNPs in coding sequences of canonical transcripts).

To exclude potential bias from rare SNPs, we filtered to retain SNPs with a minor allele frequency (MAF) of at least 0.01, resulting in a total of 74,442 SNPs. To focus on SNPs more likely to have a functional effect in planta, we only retained SNPs in genes that were expressed at >0.5 tpm in at least one tissue using data from Choulet *et al.* (2014). We excluded SNPs in regions that He *et al.* (2019) identified as being under environmental adaptation, improvement selection or within a selective sweep, as positive and purifying selection have similar impacts on nucleotide diversities in populations (Cvijovic *et al.* 2018). Introgressed sites were also excluded as they would have had a different demographic history compared to the remainder of the genome. Synonymous sites that had more than one annotation were excluded from analyses. This left 16,119 SNPs (1,020 TF, 15,099 non-TF).

We categorized the SNPs according to variant effect (stop gained, missense, and synonymous). Missense mutations were further categorized as deleterious or tolerated according to their SIFT prediction (Sim *et al.* 2012). A SIFT score of ≤0.05 is predicted to be deleterious, affecting the protein phenotype and a score >0.05 is predicted to be tolerated, not affecting phenotype.

Per site nucleotide diversity was estimated using VCFtools c0.1.16 (Danecek *et al.* 2011). Mann–Whitney tests were used to compare the TF and non-TF nucleotide site diversity distributions. Mutation load was estimated by calculating the number of homozygous alternate alleles for each site type, divided by the summed lengths of all the canonical transcripts for TFs and non-TFs separately. A linear regression with mutation load as the response and the category of sites (stop gained, deleterious missense, tolerated missense, synonymous) and the group of genes (TF and non-TF) was fitted, and an ANOVA was performed to test for the significance of the fixed effects. Furthermore, a Tukey’s test was used to compare TFs and non-TFs for each site category. Individuals with extreme mutation loads were classed as those with loads in the 2.5% tails in any of the distributions. For the TF families plot, we excluded SNPs which are only represented in individuals with extreme mutation loads. We plotted the proportion of SNPs by variant effect for TF families containing more than 10 triads and ≥5 SNPs and for non-TFs.

## Results

### TFs homoeologs are retained across polyploidization events more frequently than non-TFs

**Fig. 1.**
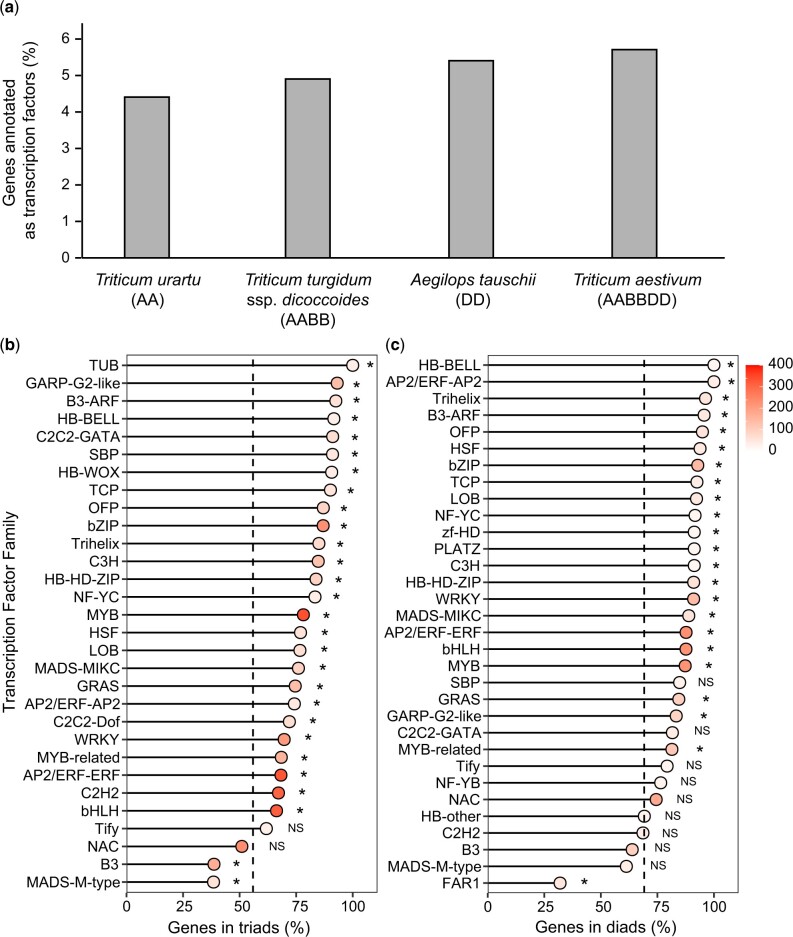
TF genes in *T. aestivum* and ancestral species. a) Percentage of genes annotated as TFs in hexaploid *T. aestivum* and the tetraploid and diploid ancestral species. b) Percentage of genes in triads in *T. aestivum* TF families with >10 triads and c) percentage of genes in diads in *T. turgidum* ssp. *dicoccoides* TF families with >10 diads. In (b) and (c), the dotted black line indicates the mean value for non-TFs and asterisks (*) denote families which are significantly different from non-TFs (Fisher’s exact test, *P* < 0.05, FDR corrected for multiple testing). NS, nonsignificant. The fill saturation inside the dots indicates the number of genes in the TF family.

To explore TF evolution and conservation in polyploid wheat, we annotated TFs in the hexaploid *T. aestivum* (AABBDD), the tetraploid ancestor *T. turgidum* ssp. *dicoccoides* (AABB) and the diploid ancestral species *T. urartu* (AA) and *A. tauschii* (DD; Supplementary Tables 1–4). We found that the percentage of genes in the genome which were annotated as TFs was 4.4% in diploid *T. urartu*, 4.9% in tetraploid *T. turgidum* ssp. *dicoccoides*, 5.4% in diploid *A. tauschii*, and 5.7% in hexaploid *T. aestivum* (Fig. 1a). This supports the hypothesis that TFs are preferentially retained, compared to other types of genes, in polyploid wheat. The retained TFs were distributed similarly across the genomes in tetraploid (50.4% on A genome, 49.6% on B genome) and hexaploid wheat (33.7% on A genome, 33.1% on B genome, and 33.3% on D genome), consistent with previous reports that wheat does not show biased subgenome fractionation associated with preferential loss of genes associated with one subgenome [International Wheat Genome Sequencing Consortium (IWGSC) 2014].

We hypothesized that the higher proportion of TF genes in polyploid wheat compared to their wheat progenitors was due to the preferential retention of TF homoeologs, whilst other types of genes were less often retained with all homoeologs. Consistent with this hypothesis, we found that in polyploid wheat, TFs were more frequently present with all homoeologs than other types of genes. Across TF and non-TF genes in hexaploid *T. aestivum*, 56.7% of genes are in triads with a single A homoeolog, a single B homoeolog, and a single D homoeolog. TF genes were more commonly found in triads with 70.5% of TFs in triads, compared to other types of genes (55.9% in triads; *P* < 0.001, Fisher’s exact test). This enrichment for triads was observed in nearly all TF families (Fig. 1b, Supplementary Fig. 1). Similar trends were observed in tetraploid *T. turgidum* ssp. *dicoccoides*. Across TF and non-TF genes, 69.8% of genes in the tetraploid were in diads with a single A homoeolog and a single B homoeolog, but this figure rose to 82.5% of TFs, compared to 69.2% of other types of genes (*P* < 0.001, Fisher’s exact test). The enrichment for diads was common to most TF families (Fig. 1c, Supplementary Fig. 2).

In general, TF families with a lower percentage of triads in hexaploid wheat already had a lower proportion of diads in tetraploid. For example, the B3 and MADS-M-type families had fewer triads/diads in both wheat species than non-TF genes, with tetraploid having 63.9% and 61.3% of genes for the B3 and MADS-M-type family in diads, respectively, and hexaploid having 38.7% and 38.5% of genes in triads, respectively (Fig. 1, b and c). The NAC TF family, which is one of the largest TF families in wheat, is one of the less well-retained TF families in tetraploid (74.5% of genes in diads), although this is still higher than for non-TFs. However, in hexaploid wheat only 51.0% of NACs are in triads which are lower than for non-TFs. After accounting for gene loss in tetraploid wheat, the B3, MADS-M-type, and NAC families in hexaploid wheat still had significantly fewer genes in triads (60.5%, 62.8%, and 68.5%, respectively) than non-TFs (80.8%; FDR adjusted *P* < 0.001 Fisher’s exact test). This indicates that homoeolog loss in specific TF families occurred across both polyploidization steps and was not solely due to pre-existing gene loss in the tetraploid.

### Differential conservation of TF families as triads is correlated with expression level and tandem duplications

To understand why certain TF families are more prone to homoeolog loss, we explored two previously proposed hypotheses. The first hypothesis is that gene families which are highly expressed are more likely to be retained with homoeologous copies (Seoighe and Wolfe 1999; Freeling 2009). Secondly, we investigated whether gene families which have more tandem duplications are less likely to be retained with homoeologous copies, as predicted by the gene balance hypothesis (Birchler and Veitia 2007).

**Fig. 2.**
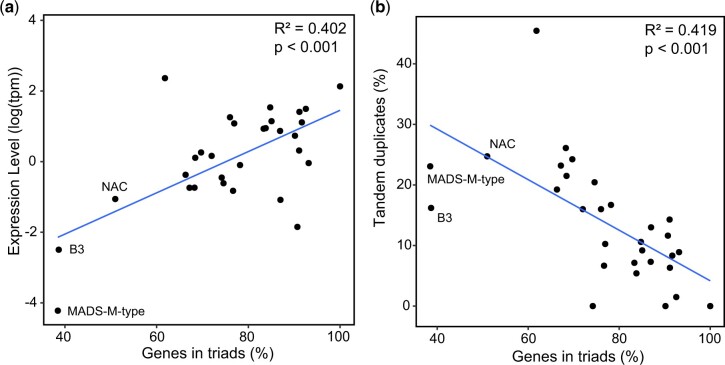
Factors explaining differential retention of homoeologs in different TF families. a) Median expression level per TF family plotted against the percentage of the TF family in triads for TF families with >10 triads. The mean expression level of each gene in tpm was calculated using 15 tissues of Chinese Spring RNA-seq data and these gene-level values were used to calculate median expression level within the TF family. b) The percentage of tandem duplicated genes within each TF family plotted against the percentage of the TF family in triads for TF families with >10 triads. TFs were considered to be tandem duplicates when they were up to ±3 genes away from each other (i.e. up to two genes in between duplicates).

To test the correlation between gene expression level and gene retention in hexaploid wheat we used RNA-seq data from 15 different tissues from Chinese Spring from a developmental timecourse (Choulet *et al.* 2014). We calculated the mean expression level for each gene across the 15 tissues and then calculated the median expression level for each TF family. Focusing on TF families with >10 triads, we found a significant positive correlation between the expression level of the TF family and the percentage of genes in the TF family which are in triads (*R*^2^ = 0.40, *P* < 0.001; Fig. 2a). This relationship also held across all TF families regardless of size, although the correlation was weaker due to small families that were outliers (*R*^2^ = 0.21, *P* < 0.001; Supplementary Fig. 3). Consistent with this relationship, the three TF families with a lower retention of homoeologs in hexaploid wheat than non-TFs (NAC, MADS-M-type, and B3) all had low median expression levels (Fig. 2a).

We also explored the relationship between tandem duplications and gene retention. Focusing on TF families with >10 triads, we found that the degree of tandem duplication in a TF family was negatively correlated with the % of triads within the TF family, consistent with the gene balance hypothesis (*R*^2^ = 0.42, *P* < 0.001, permitting up to two genes between tandem duplicated TFs; Fig. 2b). This correlation held with a more stringent criteria for tandem duplicates only permitting one gene between tandem duplicates (*R*^2^ = 0.38, *P* < 0.001; Supplementary Fig. 4a) or zero genes between tandem duplicates (*R*^2^ = 0.28, *P* = 0.003; Supplementary Fig. 4b). These relationships also held when including all TF families regardless of size, although the correlation was weaker (*R*^2^ = 0.14–0.22, *P* < 0.003) due to variability within small families (Supplementary Fig. 4, c–e). The NAC TF family that had low retention of homoeologs in hexaploid wheat had quite high levels of tandem duplication (Fig. 2b). However, the MADS-M-type and B3 TF families had lower levels of tandem duplication than the trendline across all TF families (Fig. 2b), suggesting that low expression levels (Fig. 2a) may be driving the lack of homoeolog retention in these families. Together these results indicate that different retention levels in individual TF families are associated with gene expression level and the degree of tandem duplication.

### TF triads do not show increased divergence of expression or coexpression patterns

There are several different mechanisms that can contribute to the retention of homoeologs following polyploidization. Conant *et al.* (2014) proposed a pluralist framework in which dosage effects, subfunctionalization and neofunctionalization interplay to preserve duplicated genes, in a time-dependent manner. Although transcriptomics cannot provide a definitive answer about the contributions of these different mechanisms (Conant *et al.* 2014), it can provide a starting point to understand potential mechanisms operating.

First, we used the same RNA-seq samples from 15 tissues from Chinese Spring to test whether TF triads had increased variability in homoeolog expression levels compared to non-TF triads, which would support sub- or neofunctionalization of TF homoeologs at the gene expression level leading to TF retention. We normalized the global expression of each triad so that total expression level of the triad was 1 as described in Ramírez-González *et al.* (2018), to account for differences in expression level between TFs and non-TFs. We found that the standard deviation between the expression levels of homoeologs within TF triads was not significantly different from non-TF triads in 14 out of 15 tissues (Mann–Whitney test, *P* > 0.05). Only roots at Zadoks stage 39 (flag leaf ligule just visible) had a significantly lower standard deviation between homoeolog expression levels in TF triads than in non-TF triads (median 0.093 for TF triads, 0.099 for non-TF triads, *P* = 0.036, Mann–Whitney test). Overall, the standard deviation between homoeolog expression levels was not higher in TFs than non-TFs in any tissue suggesting that sub- or neofunctionalization at the gene expression level is not different between TF and non-TF triads globally.

**Fig. 3.**
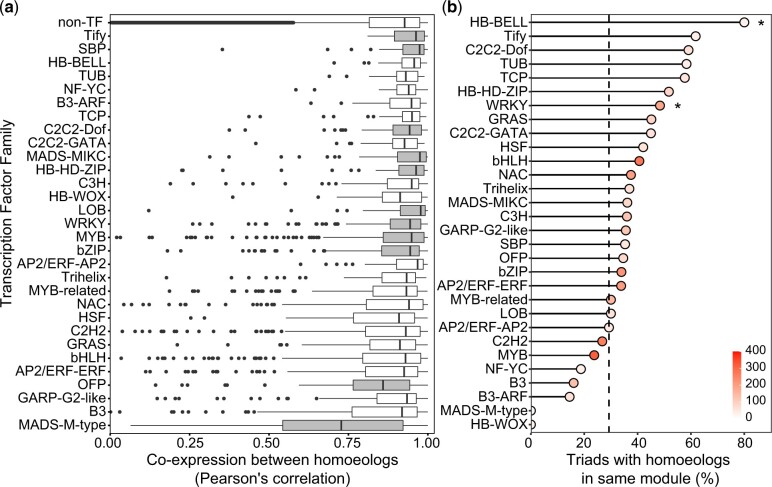
Coexpression of homoeologs within triads in TF families with >10 triads. a) Pearson’s correlation coefficient between homoeologs across 15 tissues per TF family. TF families which were significantly different to non-TFs are highlighted in gray (Mann–Whitney test, *P* < 0.05, FDR corrected for multiple testing). The correlation between non-TF homoeologs is shown in the top row. b) Homoeologs in same module in 850 sample WGCNA network per TF family. Black dotted line in (b) represents mean value of non-TFs and asterisks (*) denote families which are statistically significant different from non-TFs (Fisher’s exact test, *P* < 0.05, FDR corrected for multiple testing). The fill saturation inside the dots in (b) indicates the number of genes in the TF family.

Building upon this finding, we explored coexpression between homoeologs across different tissues. We calculated the Pearson’s correlation coefficient pairwise between homoeologs across the 15 Chinese Spring tissues. Coexpression was higher for TF triads than non-TF triads (Pearson’s correlation coefficient 0.938 vs 0.923, *P*-value <0.001, Mann–Whitney test). Amongst TF families with over 10 triads, most TF families showed higher homoeolog coexpression than non-TFs, and the differences were significant for nine TF families (Fig. 3a). Two TF families have significantly lower homoeolog coexpression than non-TFs (OFP and MADS-M-type, Fig. 3a) The trend for higher coexpression within TF families than non-TFs was also observed in TF families with fewer than 10 triads (Supplementary Fig. 5).

As an alternative measure of triad coexpression, we explored a previously generated coexpression network made using WGCNA across 850 wheat RNA samples from diverse tissues and developmental stages [Langfelder and Horvath 2008; International Wheat Genome Sequencing Consortium (IWGSC) 2018]. We found that TF homoeologs were more frequently assigned to the same coexpression module than non-TF homoeologs (35.5% vs 29.3%, *P* < 0.001, Fisher’s exact test), consistent with our Pearson’s correlation approach. A higher level of coexpression in TFs than non-TFs was consistent across most TF families in this WGCNA-based approach although the difference was only statistically significant in a few families after adjustment for multiple testing (Fig. 3b and Supplementary Fig. 6). TF families which showed higher coexpression were quite consistent with both measures of coexpression, e.g. Tify and WRKY, whilst some other families such as MADS-M-type TFs had lower coexpression using both measures (Fig. 3). Overall, we did not find support for higher levels of sub- or neofunctionalization at the expression or coexpression level in TF triads than in non-TFs, suggesting that other mechanisms such as dosage may be important for TF retention.

### Reduced deleterious mutation load in TF triads compared to non-TFs

**Fig. 4.**
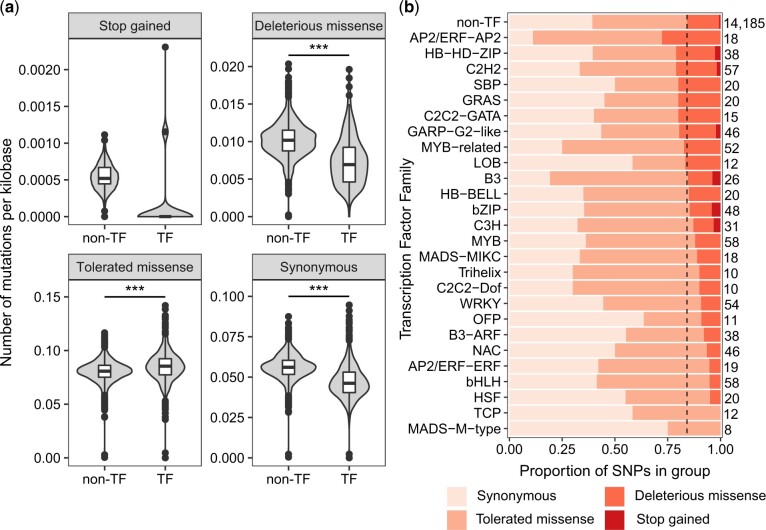
TFs accumulate fewer harmful mutations than non-TFs. a) Mutational burden in TFs compared to non-TFs for different categories of variants in 811 individuals. Mutational burden was calculated as the number of homozygous mutations of each type per individual scaled by the total length of all canonical transcripts for TFs and non-TFs. The total number of polymorphic sites analyzed for TFs and non-TFs were: stop gained (TF = 7, non-TF = 107), deleterious missense (TF = 125, non-TF = 2,277), tolerated missense (TF = 474, non-TF = 6,723), synonymous (TF = 414, non-TF = 5,989). *** indicates *P* < 0.001 following a Tukey’s test comparing the different classes of variants. b) The proportion of SNPs by variant effect in TF families containing >10 triads and ≥5 SNPs. These SNPs are in genes expressed in at least one tissue and have MAF ≥0.01. The number of SNPs in each group is shown to the right of the bars. TF families are sorted according to the proportion of deleterious missense plus stop gained SNPs, and non-TFs are shown in the top row. The black dotted line represents the split between (synonymous + tolerated missense) and (deleterious missense + stop gained) SNPs in non-TFs.

To investigate how TFs evolve in wheat populations, we explored SNPs in TFs and non-TFs using an exome capture dataset of 811 diverse hexaploid wheat cultivars and landraces (He *et al.* 2019). We hypothesized that TF triads would accumulate fewer deleterious mutations than non-TF triads, which would be consistent with their preferential retention during polyploidization. We did not observe significant differences in the distribution of deleterious or synonymous nucleotide site diversities, estimated using π, between TFs and non-TFs (Supplementary Fig. 7). π is low when allele frequency is low or high (Supplementary Fig. 8) and, therefore, it does not capture the deleterious load burden in TFs and non-TFs. To identify the mutational burden, we calculated the number of homozygous deleterious and synonymous mutations in TF and non-TF triads. The numbers of homozygous mutations per individual scaled by the total length of all canonical transcripts differ between TF and non-TF genes (ANOVA, *F* = 66.5, df = 1, *P* < 0.001). There were 32.0% fewer deleterious missense mutations per kilobase in TFs compared to non-TFs (Fig. 4a; *P* < 0.001, Tukey’s test). Frequencies of homozygous stop gained mutation were not significantly different between TFs and non-TFs. However, only seven stop gained mutations were detected in TFs making the comparison underpowered. There were 5.7% more tolerated missense mutations and 17.6% fewer synonymous mutations per kilobase in TFs compared to non-TF genes. As sites occurring in regions associated with adaptation, introgression, or domestication were removed, the lower synonymous site diversity and load in TFs likely reflects background selection.

To explore the distribution of SNP effects across TF families, we plotted the proportion of SNPs of different effects in the coding sequence of TFs in families containing >10 triads and ≥5 SNPs (Fig. 4b). Seventeen out of 26 TF families had fewer deleterious missense plus stop gained SNPs relative to non-TFs, while nine had more. The lowest proportion of deleterious plus stop gained SNPs was found in the MADS-M-Type (0.0%), TCP (0.0%), and HSF (5.0%) families, and the highest proportion in the AP2/ERF-AP2 (27.8%), HB-HD-ZIP (21.1%), and C2H2 (21.1%) families. Overall TF families vary widely in the level of deleterious polymorphism in triads.

## Discussion

### TF retention is observed in both polyploidization steps in wheat

In this study, we found that across recurrent polyploidization steps, wheat retains TF homoeologs more frequently than non-TF homoeologs. This complements previous studies where TF retention was observed in paleopolyploid events (>5 mya), neopolyploid events (∼7,500–12,500 years ago) and recent polyploidization events (<100 years ago; Buggs Richard et al. 2012; Li *et al.* 2016; Zhang *et al.* 2021). Together, these results suggest that TF retention is observed regardless of the time since polyploidization and that retention is cumulative over polyploidization steps. Consistent with the gene balance hypothesis, the degree of retention between different TF families was associated with both expression level and the degree of tandem duplication, demonstrating that even within a functional class this hypothesis can make accurate predictions.

### Lower gene expression divergence between TF homoeologs

Using the gene expression data from 15 tissues, we found that overall TFs in wheat do not show divergent patterns between homoeologs at the expression or coexpression level which differs from results in other species (Liang and Schnable 2018). For example, TF duplicates formed by paleopolyploidization events in Arabidopsis during the α, β, and γ events (all >15 mya) and maize (5–12 mya) tended to have divergent expression patterns with one copy retaining ancestral (prior to WGD event) expression patterns, whilst the other diverged in expression patterns (Pophaly and Tellier 2015; Panchy *et al.* 2019). In Arabidopsis, the copy with divergent expression tended to have more novel cis-regulatory sites, suggesting that neofunctionalization might be happening (Panchy *et al.* 2019). One reason for the lower divergence in TF homoeolog expression patterns in hexaploid wheat is that the polyploidization event is much more recent than previously studied paleopolyploidization events. Alternatively, this difference may be because wheat does not show biased genome fractionation (Juery *et al.* 2021) and has negligible global subgenome expression dominance (Harper *et al.* 2016; Ramírez-González *et al.* 2018) unlike many other studied allopolyploid species.

Although our global analysis did not show divergent patterns of expression, we found that homoeolog coexpression levels were variable between TF families. It was previously reported that a subset of triads that are dynamic in their homoeolog expression between tissues have divergent cis-regulation (Ramírez-González *et al.* 2018) suggesting that a small number of these changes may already be occurring in wheat. Given the highly similar expression and coexpression patterns observed in most TF families, it seems more likely that maintenance of gene dosage underlies TF retention in wheat, although sub- or neofunctionalization of homoeolog expression may play a role in homoeolog retention in TF families that show weaker coexpression. It would require further study to establish whether cis-regulatory changes might explain differences in coexpression between TF families.

### Deleterious variation is reduced in TF triads indicating purifying selection

We found that hexaploid wheat TF triads have fewer deleterious missense mutations than non-TF genes. This could reflect selection against gene loss, selection against neofunctionalization, or both, i.e. purifying selection for retaining each homoeolog in its original function. Our results are consistent with Brassica allotetraploids in which TFs were enriched amongst genes without any missense mutations compared to their diploid ancestors (Zhang *et al.* 2021). However, this contrasts with paleopolyploid TF homoeologs in Brassicas which have more frequent missense mutations than other genes (Zhang *et al.* 2021). This apparent contradiction could be explained by findings from 37 angiosperm species in which TFs were enriched amongst genes that were retained in duplicate for millions of years after WGD but eventually returned to singleton status (Li *et al.* 2016). Therefore, Zhang *et al.* (2021) hypothesized that selection pressure on TFs is dynamic, with a strong purifying selection for a short period after polyploidization (hence reduced missense mutations observed in hexaploid wheat), followed by a period with lower selection pressure once the target genes are lost through the diploidization process. Further studies will be needed on polyploids that formed 1–5 million years ago to test this hypothesis.

### Differences between TF families

We found that TF families showed quantitative variation in their degree of diad and triad retention, degree of tandem duplication, coexpression within triads, and deleterious SNP variation. While most TF families fell within a continuum of variation, the MADS-M-type family was an outlier in several analyses with the lowest percentage of genes in triads (Fig. 1b) and exceptionally low coexpression levels (Fig. 3) out of all 30 TF families with >10 triads. Selection to retain MADS-M-type genes appears to have been weaker than that for other TF families at both polyploidization steps, with a gradual decrease from tetraploid to hexaploid wheat, which parallels lower retention after the most recent paleopolyploidization event in Arabidopsis (Freeling 2009). This low retention is consistent with previous reports that genes in the MADS-M-type family experience a high rate of birth-and-death evolution, low positional stability within chromosomes of related species, weaker purifying selection and are less conserved between species than MADS-MIKC genes (Nam *et al.* 2004; Freeling *et al.* 2008). Counter-intuitively we found that MADS-M-type triads that are retained, are highly conserved between wheat cultivars with no stop gained mutations or deleterious missense SNPs. One explanation for the contradiction of low MADS-M-type retention during polyploidization but high conservation within hexaploid wheat cultivars could be due to their role in maintaining speciation boundaries and importance in plant reproduction (Masiero *et al.* 2011). Alternatively, the apparent high level of conservation may be due to the low number of SNPs in the MADS-M-type family included in our analysis, which is a consequence of the low level of expression of many of these genes. The MADS-M-type family contrasts strongly with the related MADS-MIKC family which behaves more similarly to other TF families and is frequently retained as triads, consistent with a previous study on the MADS-MIKC family (Schilling *et al.* 2020). While not the focus of this study, there is also likely to be extensive variation within the non-TF genes which consist of a highly heterogeneous set of genes for both function and propensity to be retained as triads.

### Implications for wheat breeding

In general, we found that TF triads are retained in hexaploid wheat and have relatively few deleterious mutations, consistent with negative consequences to changing TF dosage. However, mutations in TFs which affect dosage, such as dominant mutations, have been very important in wheat breeding for their beneficial agronomic effects, for example to adapt flowering time (e.g. *VRN1* and *PPD1*; Yan *et al.* 2003; Beales *et al.* 2007). Therefore, there is the potential to further alter gene dosage of TFs for agronomic benefit. It has been proposed that TFs with lower coexpression across tissues, termed dynamic genes (Ramírez-González *et al.* 2018), have fewer common targets (Harrington *et al.* 2020) which might release selective pressure to retain all three copies to maintain genetic balance. Therefore, one promising avenue to influence wheat phenotype by altering TF function would be to focus on TF triads with high coexpression which are more likely to have stronger phenotypic consequences if just one copy is removed. Conversely, one could focus on TF triads with low coexpression because the three homoeologs may have diverged in function, and therefore mutating one copy might lead to a phenotypic effect due to limited genetic redundancy. The recent developments in wheat functional genomics such as TILLING and gene editing now make it possible to test the effectiveness of these strategies (Krasileva *et al.* 2017; Gao 2021).

Although the possibility to alter the sequence of one homoeolog and induce a phenotypic change in wheat is attractive, there is evidence that this will not be effective for all TFs. For example, *VRN1* null mutants in a tetraploid background flower much later than wild-type plants and single mutants in the A homoeolog have an intermediate flowering time; however, single mutants in the B homoeolog of *VRN1* do not differ in their flowering time to WT (Chen and Dubcovsky 2012). A similar lack of phenotype in a single mutant was observed for *NAM2* mutants which senesce at a similar time to wild type, whereas null mutants had a significant delay in senescence (Borrill *et al.* 2019). Therefore, there will still be a need for detailed functional characterization of individual TFs, although this could be guided by predictions informed by the gene balance hypothesis.

## Data availability

The data that support the findings of this study are available in the Supplementary material of this article and from public repositories mentioned in the methods section. Scripts and input files are available at https://github.com/Borrill-Lab/TF_Triads.

Supplemental material is available at *G3* online.

## Supplementary Material

jkac147_Figure_S1

jkac147_Figure_S2

jkac147_Figure_S3

jkac147_Figure_S4

jkac147_Figure_S5

jkac147_Figure_S6

jkac147_Figure_S7

jkac147_Figure_S8

jkac147_Supplementary_Tables

jkac147_List_of_supplemental_materials
