# Supplementary material for: Transcription factor retention through multiple polyploidization steps in wheat
Source: G3 (Bethesda). 2022 Jun 24;12(8):jkac147. doi: 10.1093/g3journal/jkac147 (PMC9339333; doi:10.1093/g3journal/jkac147)
Supplement: jkac147_Figure_S2 [file jkac147_figure_s2.pdf]

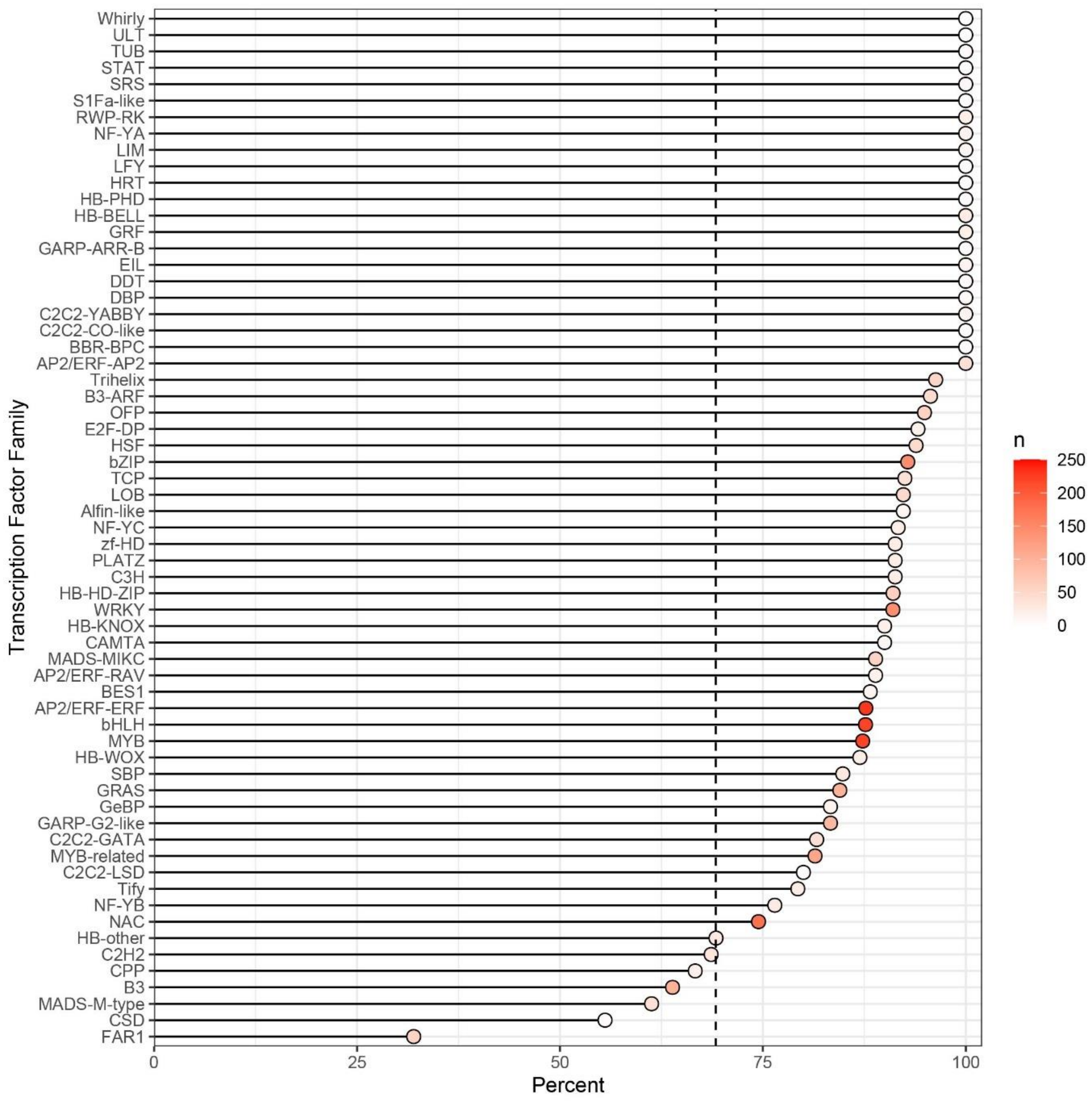

Figure S2. Percentage of genes in diads in *T. turgidum* ssp. *dicoccoides* T transcription factor (TF) families. The dotted black line indicates the mean value for non-transcription factors. The fill colour of the dots indicates the number of genes in the TF family.
