# Supplementary material for: Transcription factor retention through multiple polyploidization steps in wheat
Source: G3 (Bethesda). 2022 Jun 24;12(8):jkac147. doi: 10.1093/g3journal/jkac147 (PMC9339333; doi:10.1093/g3journal/jkac147)
Supplement: jkac147_Figure_S3 [file jkac147_figure_s3.pdf]

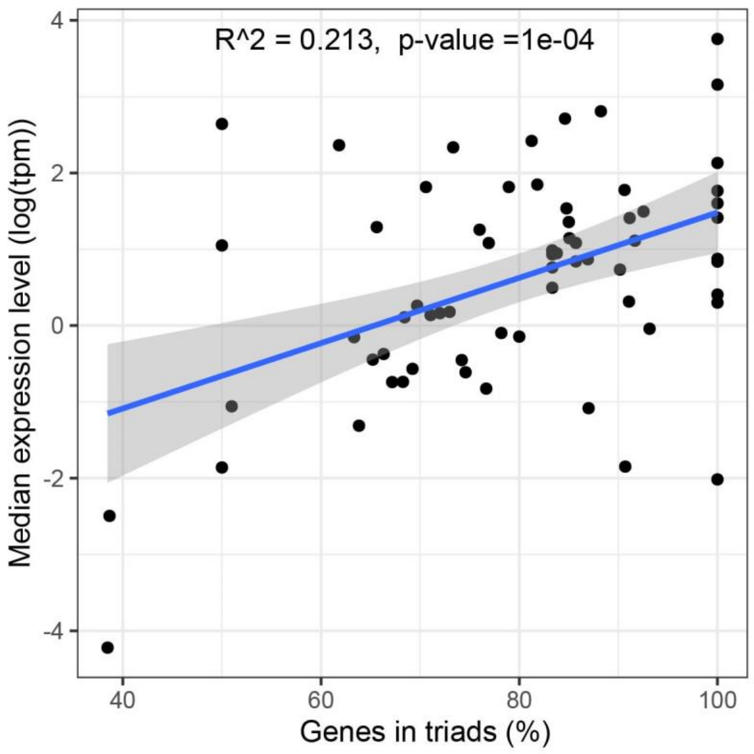

Figure S3. Median expression level per transcription factor (TF) family plotted against the percentage of the TF family in triads. The mean expression level of each gene was calculated using 15 tissues of Chinese Spring RNA-seq data and these gene level values were used to calculate median expression level within the TF family.
