# Supplementary material for: Transcription factor retention through multiple polyploidization steps in wheat
Source: G3 (Bethesda). 2022 Jun 24;12(8):jkac147. doi: 10.1093/g3journal/jkac147 (PMC9339333; doi:10.1093/g3journal/jkac147)
Supplement: jkac147_Figure_S4 [file jkac147_figure_s4.pdf]

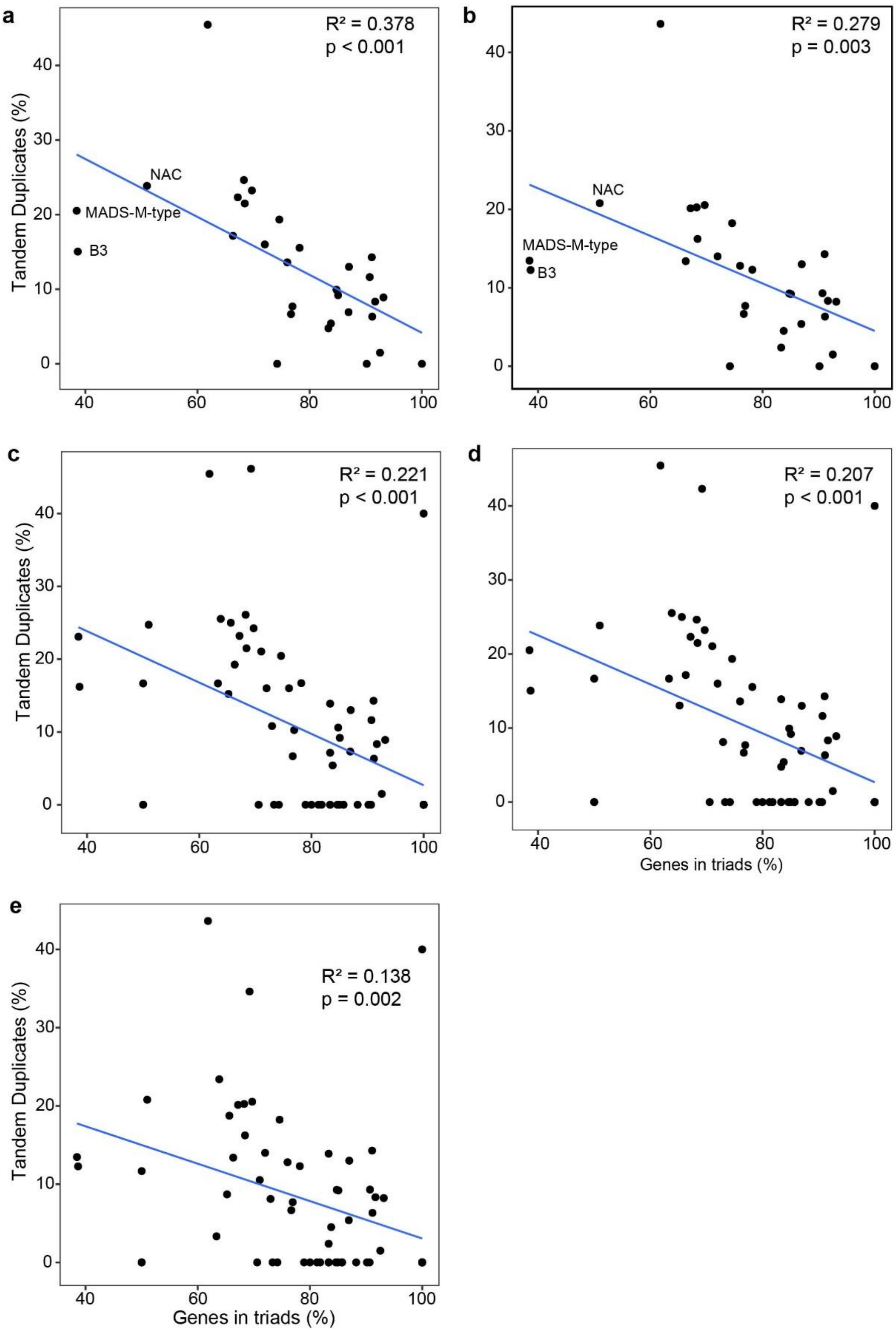

Figure S4. Relationship between tandem duplication within each transcription factor (TF) family and percentage of the TF family in triads. a) and b) show TF families with >30 genes in triads. c), d) and e) show all TF families. a) and d) Define tandem duplicates as up to ±2 genes away from each other (i.e. with 0 or 1 gene between duplicated TFs). b) and e) Define tandem duplicates as ±1 genes away from each other (i.e. with 0 genes between duplicated TFs). c) Defines tandem duplicates as up to ±3 genes away from each other (i.e. with 0, 1 or 2 genes between duplicated TFs).
