# Supplementary material for: Transcription factor retention through multiple polyploidization steps in wheat
Source: G3 (Bethesda). 2022 Jun 24;12(8):jkac147. doi: 10.1093/g3journal/jkac147 (PMC9339333; doi:10.1093/g3journal/jkac147)
Supplement: jkac147_Figure_S5 [file jkac147_figure_s5.pdf]

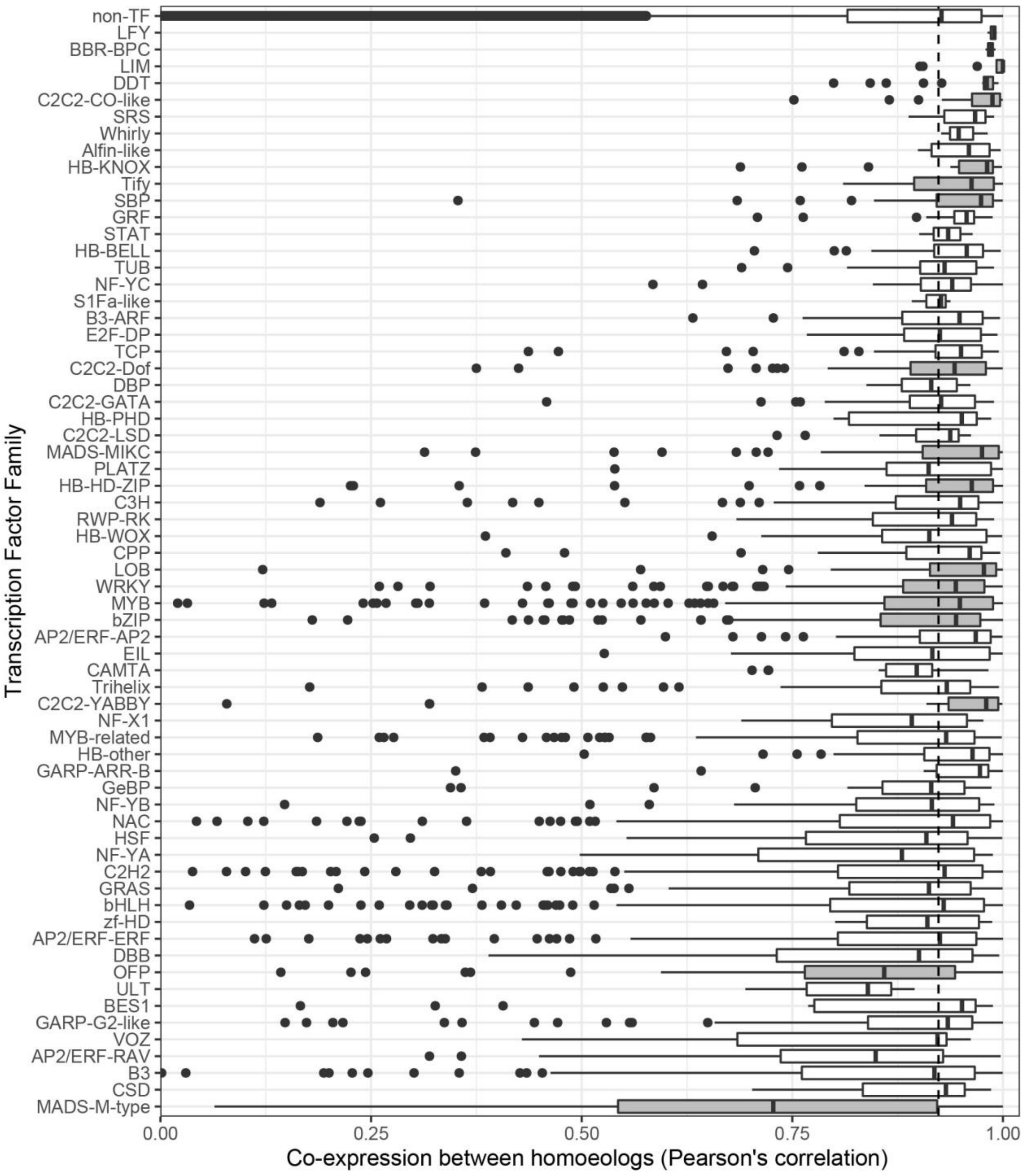


**Figure S5.** Pearson’s correlation coefficient between homoeologs across 15 tissues per transcription factor (TF) family. TF families which were significantly different to non-TFs are highlighted in grey (Mann-Whitney test, $p<0.05$, FDR corrected for multiple testing). The correlation between non-TF homoeologs is shown in the top row and the dotted vertical black line represents the median value for non-TFs.
