# Supplementary material for: Transcription factor retention through multiple polyploidization steps in wheat
Source: G3 (Bethesda). 2022 Jun 24;12(8):jkac147. doi: 10.1093/g3journal/jkac147 (PMC9339333; doi:10.1093/g3journal/jkac147)
Supplement: jkac147_Figure_S6 [file jkac147_figure_s6.pdf]

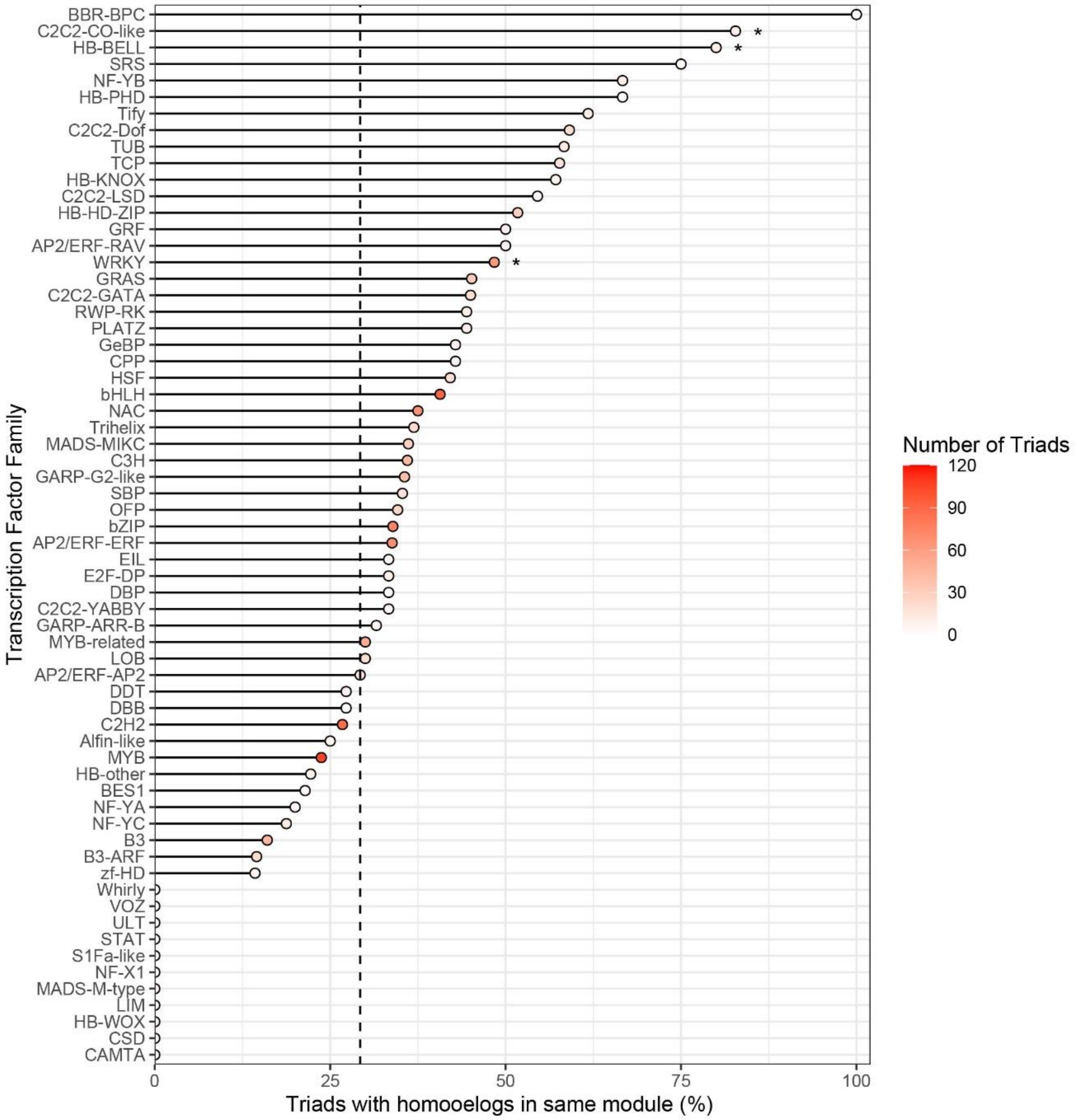

Figure S6. Homoeologs in same module in 850 sample WGCNA network per transcription factor (TF) family. Black dotted line represents mean value of non-TFs and asterisks (*) denote families which are statistically significant different from non-TFs.
