# Supplementary material for: Transcription factor retention through multiple polyploidization steps in wheat
Source: G3 (Bethesda). 2022 Jun 24;12(8):jkac147. doi: 10.1093/g3journal/jkac147 (PMC9339333; doi:10.1093/g3journal/jkac147)
Supplement: jkac147_Figure_S8 [file jkac147_figure_s8.pdf]

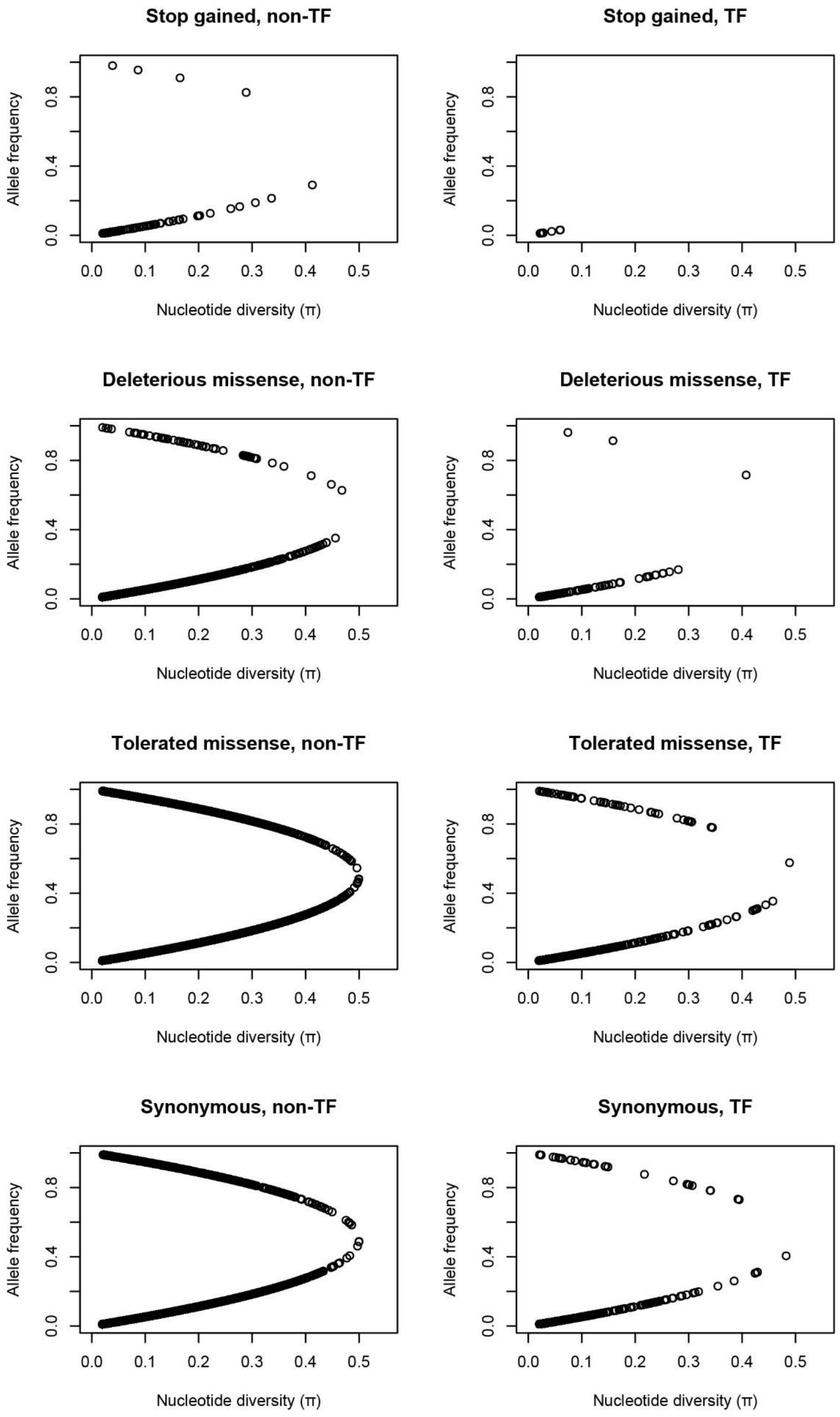

Figure S8. Association between per-site nucleotide diversity (π) and allele frequency for transcription factors (TF) and background genes (non-TF).
