# Supplementary material for: Transcription factor retention through multiple polyploidization steps in wheat
Source: G3 (Bethesda). 2022 Jun 24;12(8):jkac147. doi: 10.1093/g3journal/jkac147 (PMC9339333; doi:10.1093/g3journal/jkac147)
Supplement: jkac147_List_of_supplemental_materials [file jkac147_list_of_supplemental_materials.docx]

## Supporting Information

Table S1. Genes in *Triticum aestivum* (hexaploid wheat) assigned into transcription factor families and homoeologous groups.

Table S2. *Aegilops tauschii* genes in transcription factor families.

Table S3. *Triticum urartu* genes in transcription factor families.

Table S4. Genes in *Triticum turgidum* ssp. *diccocoides* (tetraploid wheat) assigned into transcription factor families with homoeolog information.

Figure S1. Percentage of genes in triads in *Triticum aestivum* transcription factor (TF) families.

Figure S2. Percentage of genes in diads in *Triticum turgidum* ssp. *dicoccoides* transcription factor (TF) families.

Figure S3. Median expression level per TF family plotted against the percentage of the transcription factor (TF) family in triads.

Figure S4. Relationship between tandem duplication within each TF family and percentage of the transcription factor (TF) family in triads.

Figure S5. Pearson’s correlation coefficient between homoeologs across 15 tissues per transcription factor (TF) family.

Figure S6. Homoeologs in same module in 850 sample WGCNA network per transcription factor (TF) family.

Figure S7. Distribution of per-site nucleotide diversity (π) for transcription factors (TF) and background genes (non-TF).

Figure S8. Association between per-site nucleotide diversity (π) and allele frequency for transcription factors (TF) and background genes (non-TF).
